# Renal Artery Stenosis and Mid-Aortic Syndrome in Children—A Review

**DOI:** 10.3390/jcm13226778

**Published:** 2024-11-11

**Authors:** Jakub Pytlos, Aneta Michalczewska, Piotr Majcher, Mariusz Furmanek, Piotr Skrzypczyk

**Affiliations:** 1 Student Scientific Group at the Department of Pediatrics and Nephrology, Medical University of Warsaw, 02-091 Warsaw, Poland; 2 Department of Pediatric Radiology, Medical University of Warsaw, 02-091 Warsaw, Poland; 3 Department of Pediatrics and Nephrology, Medical University of Warsaw, 02-091 Warsaw, Poland

**Keywords:** renal artery stenosis, renovascular hypertension, kidney disease, fibromuscular dysplasia, Takayasu arteritis

## Abstract

**Background:** Renal artery stenosis (RAS) and mid-aortic syndrome (MAS) are significant yet under-recognized causes of pediatric hypertension. RAS is characterized by the narrowing of the renal arteries, while MAS involves the stenosis of the abdominal aorta along with its associated vessels. The etiologies of RAS and MAS often involve genetic factors and acquired conditions such as fibromuscular dysplasia and Takayasu arteritis, contributing to their complex clinical presentations. Despite advancements in diagnostic imaging, challenges remain in effectively identifying these conditions. Pharmacological treatment can achieve partial blood pressure control, but it usually does not lead to complete recovery. Treatment options range from angioplasty to more definitive surgical interventions such as renal artery reimplantation and aorto-aortic bypass, tailored according to the specific pathology and extent of the disease. **Methods:** This review explores the diagnosis and management of RAS and MAS in children, highlighting the necessity for early detection and showcasing the evolving landscape of treatment. **Conclusions:** We advocate for a multidisciplinary approach that includes advanced imaging for effective diagnosis and tailored therapy. By integrating the latest research and clinical practices, this article provides valuable insights into managing complex vascular conditions in the pediatric population, ultimately aiming to enhance the quality of life for affected individuals.

## 1. Introduction

Renal artery stenosis (RAS) and mid-aortic syndrome (MAS) constitute relatively rare but notable etiologies of arterial hypertension in pediatric populations [1,2]. RAS is a heterogeneous disease characterized by the narrowing of the renal arteries. In contrast, MAS, often referred to as abdominal coarctation, affects the abdominal aorta along with its branches, including the renal and mesenteric arteries [3,4]. Due to an infrequence in blood pressure measurements and the presumption that high values are inaccuracies, these diseases in pediatric populations are often diagnosed only after significant delays [1]. However, this trend is shifting as there has been a notable increase in the frequency of diagnoses in recent years [5].

Considering that conventional therapy typically proves ineffective in controlling the elevated blood pressure resulting from vascular obstructions, both RAS and MAS often necessitate angioplasty or a surgical intervention [4,6]. Therefore, early identification and proper treatment are crucial in preventing permanent organ damage and improving the clinical outcomes for affected patients. The complexity of these conditions requires a multidisciplinary team to ensure their comprehensive management, thereby integrating advanced imaging techniques for precise diagnosis and individualized therapeutic options to address the unique challenges posed by each case.

This review article delves into the complexities of RAS and MAS management, highlighting their significance as contributors to pediatric hypertension and the evolving landscape of their diagnosis and management in the modern era.

## 2. Epidemiology

For decades, the prevalence of arterial hypertension in children has remained almost constant at around 4% [7]. Renovascular disease (RVS) is an uncommon but important cause of childhood hypertension, accounting for 5–10% of the secondary causes of hypertension in children [1,8,9,10]. Other rare causes of renovascular hypertension include mid-aortic syndrome; although MAS represents only 0.5–2% of all cases of aortic stenosis in general, a percentage of children (20–48%) with renovascular hypertension present with stenosis of renal arteries and a narrowing of the middle-aorta [1,11]. As reported in the European Society of Hypertension guidelines, diseases of the renal vessels resulting from lesions that cause significant impairment of blood flow to the kidneys are far less common than diseases of the renal parenchyma [12].

## 3. Etiology

In contrast to adults where the main cause of RAS is from atherosclerosis, the etiologies in the pediatric population are mostly associated with various diseases [5] (Table 1).

### 3.1. Fibromuscular Dysplasia

Fibromuscular dysplasia (FMD) is a generalized vascular disease that may affect all vascular beds and result in arterial stenosis, occlusion, aneurysm, or dissection [16]. It is regarded as the major contributor to pediatric RAS in North America and Europe [3]. FMD most commonly affects the renal and cervical arteries. The pathophysiology of FMD is still unclear. In the first report on pediatric FMD from the US multicenter registry, Green R. et al. noticed that the most common signs and symptoms in children are arterial hypertension, headaches, abdominal bruits, and dizziness and, compared to adults, children are more likely to present with arterial hypertension at the time of diagnosis [17].

### 3.2. Takayasu Disease

Takayasu arteritis is a systemic inflammatory vasculitis of unknown origin that involves large- and medium-sized blood vessels, with a predilection for the aorta and its branches. The involvement of the renal arteries results in renal artery stenosis and, consequently, renovascular hypertension. In studies of children with renovascular hypertension from India and South Africa, it is the cause of the disorder in 73% and 89% of cases, whereas in Europe and North America, only 1–2 cases/million/year have been reported [3,18]. The diagnosis in the pediatric population is difficult. Symptoms in the early phases of the disease are mostly non-specific, including fever, weight loss, and fatigue. Nevertheless, elevated blood pressure may be the sole clinical presentation [19].

### 3.3. Syndromic Diseases

In children with a suspicion or a diagnosis of RAS or MAS, it is crucial to consider syndromic etiologies. Recognizing the distinctive phenotypes associated with these syndromes is instrumental in achieving a diagnosis. The presence of diffuse stenoses is also more likely when extrarenal diseases, syndromes, or vasculitides are involved. Thus, an evaluation for extrarenal involvement, specifically in craniocervical arteries, aorta, and mesenteric and coronary arteries, is advised [1]. In cases where a syndromic cause is suspected, a referral to a genetic clinic for comprehensive genetic testing is recommended. The detailed overview of renal artery stenosis-associated syndromes is provided in Table 2.

### 3.4. Mid-Aortic Syndrome

Mid-aortic syndrome (MAS) is an interesting vascular anomaly that leads to secondary arterial hypertension stemming from renal artery stenosis. This condition is distinguished by the narrowing of the abdominal aorta and its related vessels. Due to its distinctive characteristics, MAS is frequently described as an atypical coarctation or hypoplasia of the aorta [4]. Initial case series observations indicated a higher prevalence of MAS in women and individuals younger than 25 years of age [2]. Modern analyses confirm the predominance of the syndrome in children and young adults, with the average age at diagnosis reported to be 7.1 years, and some cases identified as early as the 27th week of gestational age. However, a difference in incidence based on sex was not established [4,37].

Typical MAS represents an extremely rare vascular anomaly. It presents as a segmental or diffuse narrowing of the abdominal aorta; however, in a minority of cases [3%], it primarily affects the distal part of the thoracic aorta. In the abdominal aorta, the suprarenal region is the most commonly affected site, followed by stenosis extending from suprarenal to infrarenal areas or occurring entirely in the interrenal section. An exclusively infrarenal involvement is the least frequent occurrence [11].

The involvement of renal and visceral branches is very common among MAS patients. At initial presentation, RAS occurs in 66% of cases, with about 60% of these instances being bilateral. In only 35% of cases, the stenosis is limited to the ostial region of the vessel, which indicates that the majority of patients have either prolonged or intermittent regions of narrowing, which complicates the treatment process. In 30% of cases, the superior mesenteric artery is involved, while the celiac trunk is affected in 22% of patients. Involvement of the inferior mesenteric artery is uncommon. Additionally, vascular abnormalities are observed in the retinal and cerebral regions, present in 10% and 3% of the cases, respectively [11].

Although the etiology of MAS remains unknown, it has been theorized to arise from an improper fusion of the embryonic dorsal aortas during the first month of gestation [38]. MAS may stem from acquired inflammatory diseases (17%), genetic disorders (15%), and FMD (4%), however, the majority of cases are idiopathic (61–64%) and lack a definitive underlying diagnosis [39]. In a notable study by Warejko et al., underlying mutations were identified in a significant proportion, approximately 43%, of families affected by mid-aortic syndrome (MAS) [36]. While MAS caused by a genetic disease is the type that predominantly manifests in children, it has been recognized in young adults as well. Genetic etiologies of MAS include diseases such as Williams syndrome, Alagille syndrome, neurofibromatosis type 1 (NF1), Tuberous Sclerosis, and Turner syndrome. Acquired inflammatory conditions recognized as its culprits are Takayasu arteritis, other forms of arteritis, and intrauterine infections such as rubella [2,4,20,22,30,31].

Notably, it is reported that idiopathic MAS is more frequently associated with infrarenal aortic stenosis, whereas genetic disorders tend to show a greater incidence of suprarenal aortic involvement, as well as more extensive extra-aortic involvement, including a higher incidence of RAS. Additionally, patients with an inflammatory etiology tend to exhibit a higher incidence of abdominal aortic aneurysms, as noted in 11% of MAS cases [11].

## 4. Clinical Manifestations

The presentation of renal artery stenosis in children is variable and often asymptomatic [1]. The most common finding is isolated hypertension, which is generally very high, within systolic measurements greater than 200 mm Hg, and diastolic measurements much higher than the 95th centile for age, sex, and height [40]. For teenagers aged 16 and older, the diagnostic criteria align with adult standards, requiring a systolic blood pressure of 140 mmHg or higher and/or a diastolic blood pressure of 90 mmHg or higher [12]. Approximately 26% to 70% of cases of renovascular disease manifest with arterial hypertension in an otherwise asymptomatic child [3]. In children with elevated blood pressure, secondary hypertension should always be considered and investigated [12]. The diagnosis is often delayed due to technical problems in measuring blood pressure and a low index of suspicion in children [1]. In some cases, during physical examination, a bruit can be heard over the abdomen. In FMD, bruits can sometimes be heard overlying the epigastrium, carotid arteries, and flank, and in patients with MAS, a mid-abdominal bruit is a classic finding [3,17]. Other presentations may relate to secondary effects of hypertension, including neurological (10–15%) and cardiac (7%) findings [40]. The neurological symptoms can range from headaches, seizures, and strokes to cranial nerve palsies, amblyopia such as an isolated lower motor neuron facial palsy (Bell’s palsy), or posterior reversible encephalopathy syndrome (PRES). Younger children are more likely to have cardiac findings of palpitations, bruits, or signs of congestive heart failure [1,3]. Many children with renovascular stenosis also exhibit abnormalities in other vascular beds, which may result in a variety of clinical features depending on the arteries involved [19]. Physical findings of renal artery stenosis-associated syndromes are detailed in Table 2. It needs to be highlighted that some patients with RAS or MAS with demonstrated mutations do not have a typical phenotype, which may result from mosaicism or variable gene expression [41].

To summarize, the suspicion of renovascular hypertension should be considered in any child with severe refractory hypertension that is not controlled with two or more antihypertensive drugs, especially if other suggestive findings are present, including an abdominal bruit, and when clinical symptoms indicative of vascular damage in critical organs for hypertension (central nervous system, kidneys, and heart) are present [19,42].

## 5. Laboratory Evaluations

In a patient with renovascular hypertension, a series of laboratory tests should be conducted. Increased creatinine levels, azotemia, or electrolyte derangements, such as hyponatremia, hypokalemia, and alkalosis, can suggest RAS [3].

The level of creatinine depends on the degree of stenosis in the renal arteries. In unilateral disease, the serum creatinine concentration often remains normal due to compensation by the healthy kidney, which can mask the impairment of function in this kidney [1]. However, bilateral disease can lead to decreased renal function due to hypoperfusion.

Hypokalemia, sometimes mild or transient, can be caused by activation of the renin-angiotensin-aldosterone system (RAAS), resulting in secondary hyperaldosteronism and excessive urinary potassium loss [3].

Unilateral renal artery stenosis presenting as hypertensive hyponatremic syndrome (HHS) has been infrequently reported in pediatrics, with only a few cases reported to date. The cause of this syndrome is hyperactivation of the RAAS. Activation of the RAAS in the presence of a hypertrophied contralateral kidney sets up a vicious cycle leading to natriuresis and hyponatremia, along with the usual hypokalemia and metabolic alkalosis. This unique combination of hypertension, hyponatremia, and natriuresis strongly suggests a compromised unilateral renal blood flow [43].

In urinalysis, proteinuria and glycosuria, biomarkers of kidney damage, can be observed [3].

Increased plasma renin activity or increased plasma renin concentrations together with secondary hyperaldosteronism may also be detected but have been disappointing in clinical use. These are not sensitive enough to reliably diagnose renovascular hypertension. Elevations are observed in only half of the patients and are variably affected by ethnicity, age, medications, volume status, and other variables [44]. Nevertheless, plasma renin activity remains important in identifying cases of low-renin hypertension, particularly in monogenic forms [12].

When there is a suspicion that the cause of the stenosis is associated with an inflammatory disease, inflammatory markers should be evaluated (erythrocyte sedimentation rate and C-reactive protein).

A similar situation occurs with genetic diseases. Data show that 11–60% of cases of renovascular disease are familial, with a pattern most consistent with autosomal dominant inheritance with variable penetrance [41]. Sometimes, the whole exome sequencing (WES) needs to be performed.

## 6. Radiological Imaging

The current diagnostic approach for renovascular hypertension in children lacks a single radiological imaging method that effectively rules out all causes, including RAS and MAS, with percutaneous angiography remaining the gold standard despite its invasiveness [12,45]. The remaining methods serve to assess the necessity of angiography, monitor patients, and exclude other causes of arterial hypertension.

**Figure 1:**
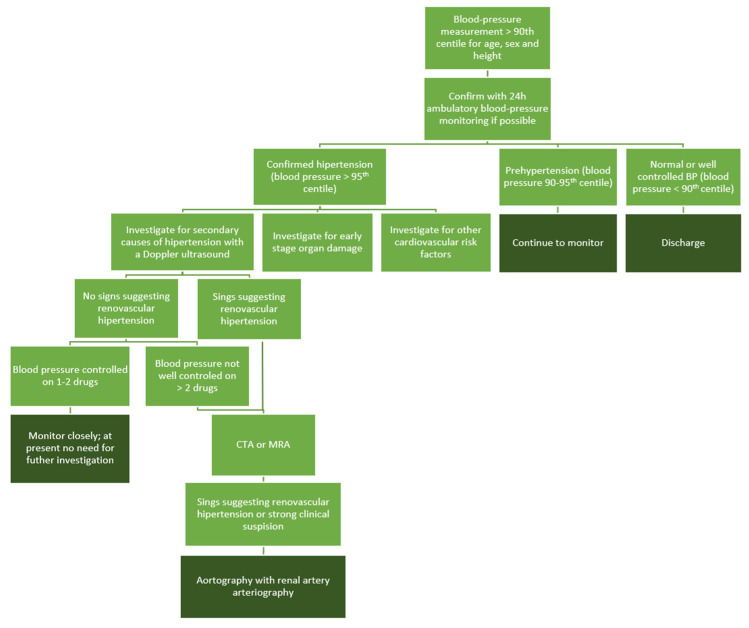
Suggested diagnostic approaches to a pediatric patient with suspected renovascular hypertension.

The efficacy of various imaging techniques is still under evaluation, aiming to establish more sensitive and specific non-invasive options. Detailed information on the sensitivity and specificity of each method is provided in Table 3. The recommended diagnostic approach is illustrated in Figure 1.

### 6.1. Ultrasound

Doppler ultrasound continues to be a preferred method for initial RAS assessment because of its safety and simplicity. It can directly visualize stenosis, identify *parvus et tardus* waveform patterns, assess pathological flow parameters, evaluate renal pathologies, and exclude certain non-vascular hypertension causes such as neuroblastoma, pheochromocytoma, Wilms’ tumor, renal cystic diseases, or dysplasia [12]. While particularly effective in older children and for aortic stenosis detection, ultrasound struggles with detecting smaller vessel stenoses, multiple renal arteries, and early branching, leading to potential false-positive and false-negative outcomes [1,46].

**Figure 2:**
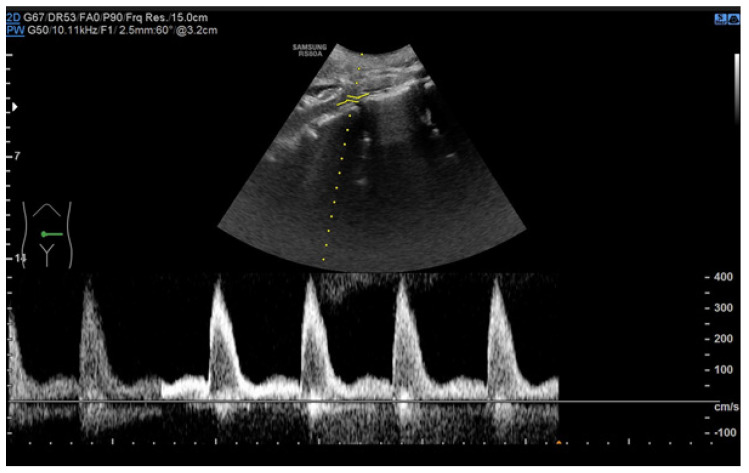
Increased maximum flow velocity (~400 cm/s) in the place of abdominal aorta stenosis; analysis of an eight-year-old ♂ patient with MAS by spectral Doppler ultrasound.

**Figure 3:**
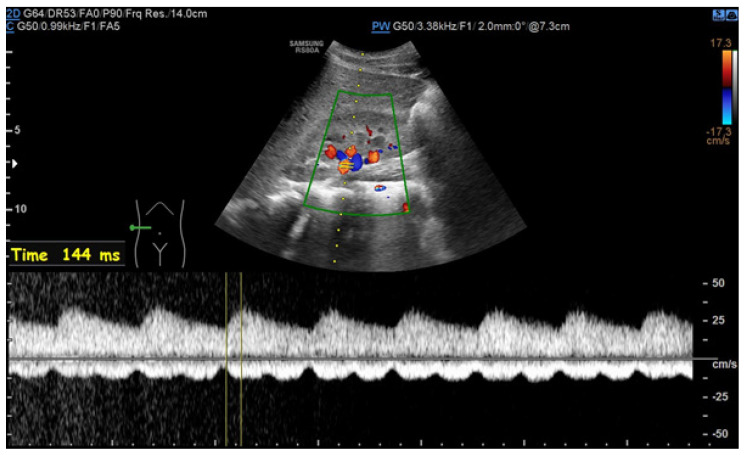
*Parvus et tardus* waveform patterns in renal artery trunk; analysis of an eight-year-old ♂ patient with MAS by spectral Doppler ultrasound.

**Figure 4:**
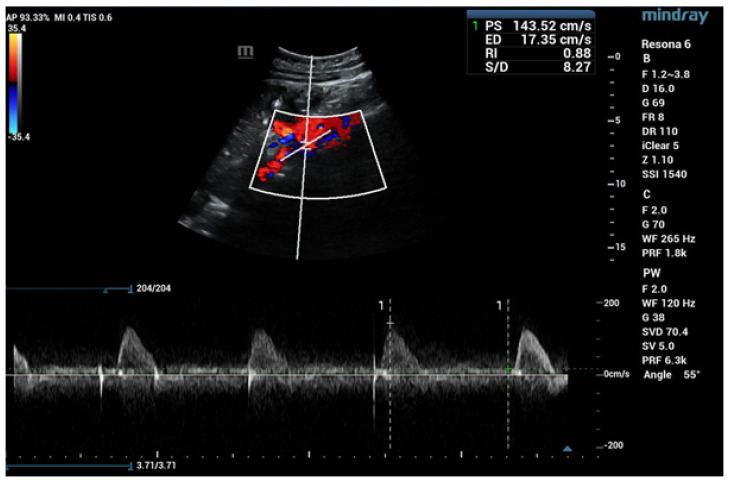
Increased maximum flow velocity (~200 cm/s) in the place of renal artery stenosis; analysis of an eleven-year-old ♂ patient with FMD by spectral Doppler ultrasound.

**Figure 5:**
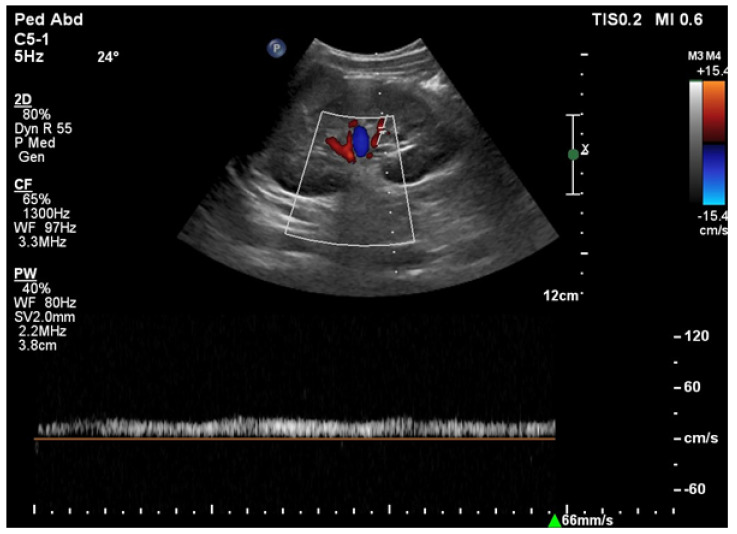
*Parvus et tardus* waveform patterns in intrarenal branches; analysis of an eleven-year-old ♂ patient with FMD by spectral Doppler ultrasound.

During ultrasound examinations to assess RAS, the Polish Society of Hypertension guidelines advocate for the evaluation of several key parameters. The renal-aortic ratio (RAR) is the ratio of the highest peak flow velocity along the renal artery, usually in the proximal segment (PSVr), to the highest peak velocity in the aorta at the level of the renal artery origin (PSVa). The maximum blood flow velocity in the renal arteries must always be considered in relation to the velocity in the abdominal aorta, where the range of the maximum velocities varies from 30 cm/s in people with circulatory failure or major atherosclerotic changes to 150 cm/s in young people with hyperkinetic circulation. It is essential to assess and compare the RAR for both renal arteries. The prolongation of acceleration time (AT) beyond 70 ms suggests flow disturbances in the prerenal segment, typically presenting unilaterally in unilateral RAS and bilaterally in conditions such as bilateral RAS, aortic stenosis, and MAS. The resistive index (RI), calculated from the peak systolic velocity (PSV) and the end-diastolic velocity (EDV) according to the formula (PSV—EDV)/PSV, is typically measured in the interlobar arteries. A RI difference exceeding 0.05 indicates unilateral stenosis, while bilateral stenosis shows a decrease in RI in both arteries below age-specific norms. Additional parameters, such as the pulsatility index (PI) and acceleration index (AI), are also valuable in the RAS assessment. The normal blood flow parameter values of the renal arteries according to the Polish Ultrasound Society guidelines are presented in Table 4 [48,49]. The recorded Doppler ultrasound examinations from our department are exhibited in Figure 2, Figure 3, Figure 4 and Figure 5.

### 6.2. Computed Tomographic Angiography (CTA)

CTA is considered to be sufficient for diagnosing renovascular hypertension [1]. It offers clear imaging of the renal arteries, their primary branches, kidney size, and parenchymal changes [3]. While sensitivity and specificity rates for CTA and MRA (Magnetic Resonance Angiography) can differ across research centers, CTA generally exhibits a slightly higher sensitivity and similar specificity compared to MRA, likely because it is unaffected by respiratory movements [46,50].

**Figure 6:**
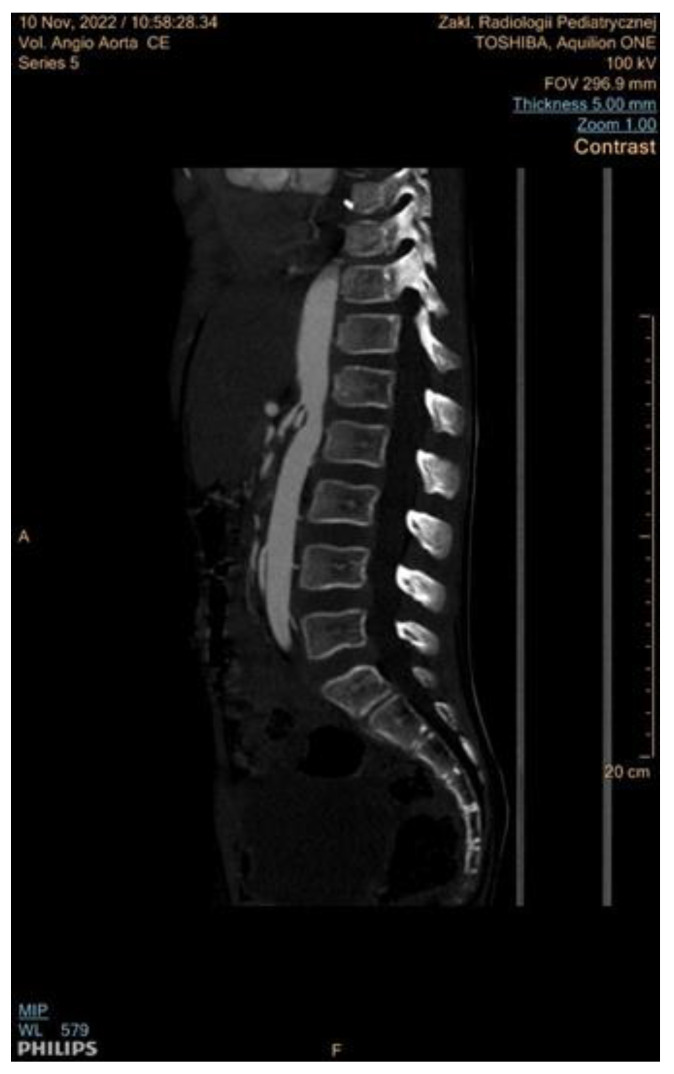
Stenosis of the abdominal aorta between the celiac trunk and renal arteries in a patient with MAS, analyzed by computed tomographic angiography.

**Figure 7:**
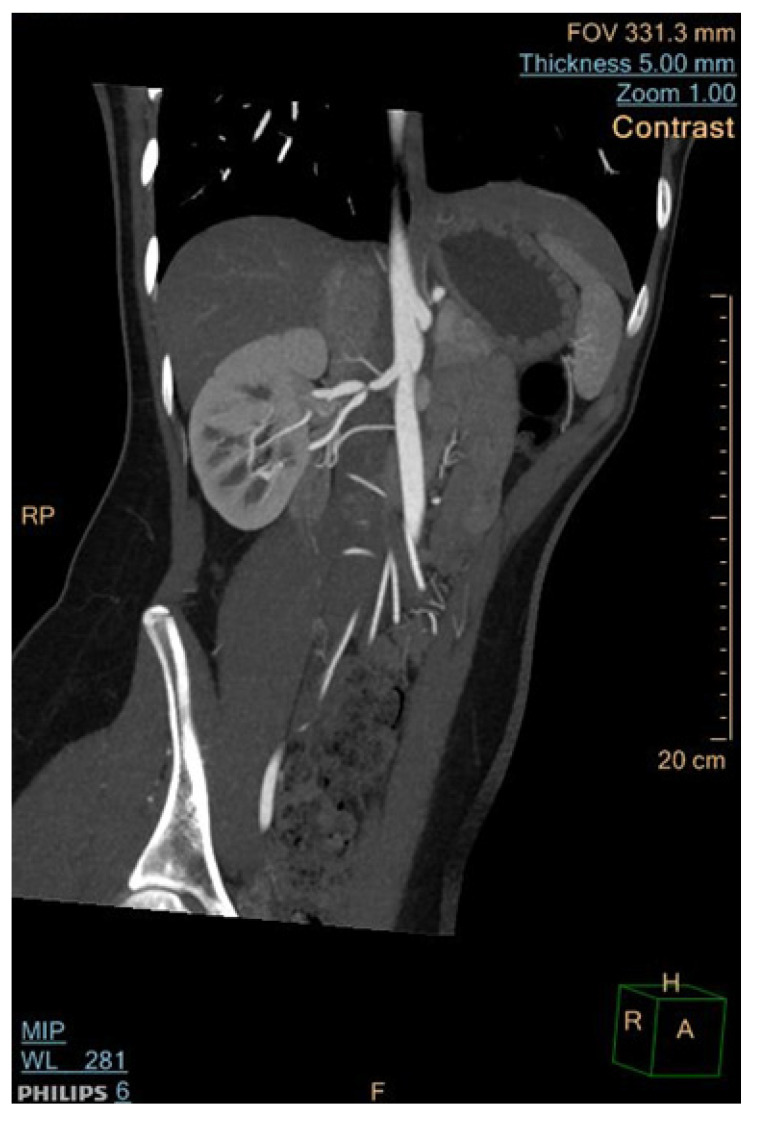
Renal artery stenosis in a patient with FMD, analyzed by computed tomographic angiography.

It remains the most effective non-invasive alternative to traditional angiography for diagnosing RAS and MAS and is particularly adept at detecting thin webs in FMD, which MRA might overlook [45]. The radiation risk associated with CTA can be reduced through the implementation of radiation minimization protocols [3,51]. CTA images recorded in our department are presented in Figure 6 and Figure 7.

### 6.3. Magnetic Resonance Angiography (MRA)

MRA is adequate for assessing the aorta and main renal arteries, offering detailed insights into renal size and blood flow without radiation exposure [1]. In pediatric populations, it remains inferior compared to CTA, owing to reduced spatial resolution, which leads to inadequate visualization of segmental renal vessels and a possible overestimation of stenosis in the main renal artery [3]. While MRA has limitations in comprehensively assessing possible causes of renovascular hypertension in children, enhancements in spatial resolution and further advances in perfusion techniques could potentially establish it as the leading non-invasive imaging technique in the future [1].

### 6.4. Renal Angiography

Catheter-based digital subtraction angiography (DSA) remains the most accurate method for diagnosing and the only technique considered capable of excluding renovascular disease in children [46,47]. Approximately 28% of vascular lesions in patients with hypertension of vascular origin are exclusively identified through angiography, underscoring its critical role in accurate diagnosis and guiding surgical decisions, particularly when non-invasive tests are inconclusive [45].

DSA is generally performed under general anesthesia in small children, while older children may undergo the procedure with only local anesthesia. For imaging that facilitates therapeutic planning, it is essential to visualize the renal arteries, establishing their number and configuration, and also the abdominal aorta with its branches and, in certain cases, the thoracic aorta [1,52].

Recent advancements such as rotational angiography are particularly useful for diagnosing complex vascular conditions in children, including large aneurysms, vessel occlusions, and complex stenoses. Similarly, intravascular ultrasound may provide details about the arterial wall not visible in conventional angiography [1,52].

## 7. Management

### Medical Management—Pharmacotherapy

The targeted treatment for renovascular hypertension involves mostly endovascular management. While pharmacotherapy can achieve partial blood pressure control, it does not lead to complete recovery. The pharmacological approach to treating renovascular hypertension depends on the type of stenosis present in either one or both renal arteries (presented in Table 5), disease etiology, patient age and size, and the clinical expertise of the institution [40,53,54]. Non-invasive treatment may present challenges related to patient adherence and adverse drug effects [5].

Continuation of medical therapy involves patients who are still undergoing evaluation for RAS, as well as those who are not candidates for angioplasty or surgical intervention due to unacceptable risk or technical infeasibility [3]. In addition, at least half of the children who undergo an interventional radiology or surgical procedure will require continued medical therapy [45].

Angiotensin-converting enzyme inhibitors and angiotensin II receptor blockers are strictly prohibited in bilateral RAS or renal artery stenosis in a solitary kidney as they can lead to acute renal failure, volume overload, and pulmonary edema. They can be very effective in unilateral RAS but their use may lead to a deterioration of function of the affected kidney. Notably, many patients with RAS require multi-drug therapy to lower blood pressure.

## 8. Endovascular Management

For the majority of pediatric renovascular hypertension cases, endovascular treatment is recommended [1]. The aim of interventional radiology is to ensure adequate renal perfusion, thereby reducing the reliance on pharmacological treatment and controlling symptoms, particularly in younger patients before surgical repair can be undertaken [46].

### 8.1. Renal Angioplasty

Percutaneous transluminal renal angioplasty (PTRA) serves as an effective treatment for pediatric RAS. Data reported by Alexander et al. from pediatric patients who underwent percutaneous transluminal angioplasty and had at least one year of follow-up blood pressure monitoring showed that the pediatric population had an overall cure rate of 36% and an improvement in blood pressure control in a further 32% of patients [55]. Other studies have reported a successful cure or amelioration of hypertension in over 50% of patients with idiopathic RAS, 36% of those with FMD, 80% of those with Takayasu arteritis, and 71% of individuals with NF1 [19,56].

In cases of arteritis, angioplasty is recommended once the inflammatory process has resolved unless there is a critical risk of renal ischemia. PTRA is typically performed via a femoral or radial approach using a vascular sheath or guiding catheter and accompanied by intra-arterial heparin administration. While arterial spasm is reported in 16.4% of procedures, dissection or perforation appear less often, and these issues are frequently not clinically significant. They can be managed through specific interventions, such as intra-arterial nitroglycerine, balloon reinflation, or, in rare instances, an emergency management with a covered stent or open surgery [1,57,58,59].

Given the complexity of PTRA, expert vascular surgical support and vigilant postoperative care, including consistent heart rate and blood pressure monitoring, are essential. Additionally, postoperative management includes antithrombotic medication, initially administering either low-molecular-weight heparin or intravenous unfractionated heparin for the first 24 h, followed by a maintenance aspirin regimen to reduce the risk of thrombosis [1,58].

### 8.2. Renal Artery Stenting

Angioplasty is often viewed as more suitable than stenting, as the stents can themselves become sites of stenosis due to either intimal hyperplasia or thrombosis. Additionally, stents have the potential to migrate, fracture, or impede future surgical revascularization efforts [52]. Therefore, stenting is typically considered only in situations of immediate recoil post angioplasty with over 50% diameter stenosis, recanalization of an occluded renal artery, or early recurrence of stenosis following a successful angioplasty [52]. In pediatric patients, balloon-expandable stents are usually favored as they allow further dilation, accommodating the growth of the patient [58]. The long-term efficacy and safety of renal artery stenting in children remain uncertain [1,46]. Available data indicate that the restenosis rate following stent placement is between 35.5% and 37% [40,60].

In pediatric renal artery stenosis, the majority of reports describe the use of bare metal stents. As an alternative approach, drug-eluting stents (DESs) have been employed in pediatric renal revascularization. DESs incorporate antiproliferative and anti-inflammatory agents to mitigate the risk of in-stent restenosis. This method is based on a similar principle to coronary artery stenting. It is worth noting that the diameters of coronary arteries in adults and renal arteries in children are similar [61].

### 8.3. Aortic Angioplasty

Angioplasty for MAS is less common than surgical intervention, with a reported occurrence rate of just 28%, yet it is still deemed viable for certain patients in order to delay surgical intervention [11]. This lower prevalence is likely attributed to the high rate of reintervention, which is reported to reach 67% at a five-year follow-up [4]. Due to the risk of thrombosis, rupture, or pseudoaneurysm, aortic angioplasty necessitates a cautious approach for effective outcomes [1]. Stenting of the abdominal aorta in children is rarely performed as it carries significant risks.

### 8.4. Ethanol Ablation

Ethanol, as a sclerosing agent, is capable of deep tissue penetration and is particularly effective in ablating extensive tissue areas [62]. When stenosis of a segmental artery is untreatable by angioplasty or reconstructive surgery, ethanol ablation is a viable, less invasive alternative to partial nephrectomy, preserving more healthy renal tissue [1,46]. Coil embolization is rarely recommended as the ischemic focus can recruit collateral supply, maintaining hypertension [1].

### 8.5. Surgery

When medical management and angioplasty are unsuccessful, surgery becomes the subsequent treatment option, as recommended by the European Society of Hypertension [12]. Additional indicators for surgical intervention are as follows: MAS, ostial or proximal lesions, long-segment stenosis, multiple stenotic areas, and aneurysms [59]. It successfully resolves hypertension in 36–70% of patients and yields significant improvements in another 26–56% [1]. In the study by Stadermann et al., normotension was achieved in 74% of children at the one-year follow-up and in 85% at the last follow-up conducted between one and ten years later, compared to only 27% who were normotensive before the surgery. Additionally, a significant reduction in the need for antihypertensive medications was observed, with the median number of drugs decreasing from four preoperatively to one at the final follow-up [63]. 

Surgery carries a significantly higher risk of major postoperative complications compared to angioplasty, with rates of 15% versus 6% [46]. The precise timing of surgery should be individually analyzed for each patient, taking into account symptom severity, medical treatment response, surgical risk assessment, and the size and age of the patient, with the ideal timing being during or after puberty [46,59,64]. Surgical options include artery reimplantation, aortorenal bypass, autotransplantation, or nephrectomy.

The preferred and most commonly practiced surgical procedure is renal artery reimplantation. This surgical approach includes an excision of the stenotic segment of the vessel followed by reimplanting the renal artery onto the aorta or one of its major branches. Consequently, the applicability of this procedure is limited by the extent and location of the vessel narrowing, making it particularly useful in cases of ostial stenosis, where the blockage occurs at the beginning of the renal artery [59,64].

The aortorenal bypass utilizes a graft, which would ideally be autogenous to accommodate growth. The use of the saphenous vein for this procedure is contraindicated because of the high risk of aneurysmal degeneration [1]. Therefore, in the absence of a suitable autogenous graft, vascular prostheses frequently emerge as the sole option, employed during or after puberty, with the objective of executing a single, definitive revascularization procedure. Other widely accepted options include utilizing the hepatic, splenic, or gastroduodenal arteries to establish collateral circulation to the kidney [1].

Renal autotransplantation involves kidney removal, an ex vivo reconstruction of the renal artery, and a reimplantation into the iliac vessels. It is an option for extensive disease or multiple renal arteries [59].

Nephrectomy is indicated when revascularization is not possible or for the removal of an atrophic kidney that contributes to hypertension [1,19]. When only a segment of the kidney has lost function, a partial nephrectomy is the preferred option.

### 8.6. Management of Mid-Aortic Syndrome

An aorto-aortic bypass from the distal thoracic aorta to the infrarenal aorta, with revascularization of all narrowed branches, is a preferred surgical approach for treating MAS. Among patients undergoing surgical interventions for MAS, it was received by 55–64% of patients, with patch aortoplasty and renal artery reconstruction alone being less common [1,59,65]. Patch aortoplasty is considered the best intervention for short-segment stenosis and in infants, factoring in their expected growth [59,65]. In children with severe stenosis of the aorta, the collateral vessel development is significant, thus aortic reconstruction should be considered exclusively in cases with incapacitating claudication or compromised lower limb development. Renal revascularization in these patients can also be accomplished using a bifurcated prosthetic graft from the supracoeliac aorta, attached via end-to-end anastomosis to one or both renal arteries [1].

### 8.7. Anticoagulation in Patients with RAS and MAS

In the pediatric population with renovascular hypertension, there are no clear indications for anticoagulant treatment. According to the international consensus, in the absence of contraindications, antiplatelet therapy (i.e., aspirin 75–100 mg daily) is indicated for all adult patients with FMD in order to prevent thrombotic and thromboembolic complications [66]. In adults with fibromuscular dysplasia (FMD), dual antiplatelet therapy before or after angioplasty is generally not recommended in most cases (90%), except in instances involving arterial dissection. Long-term single antiplatelet therapy is administered in about 60% of cases. Anticoagulants are typically restricted to bolus injections administered during surgery [67]. For pediatric patients with FMD, no specific guidelines have been established.

Furthermore, the current EULAR guidelines on Takayasu arteritis and other large vessel vasculitis treatments advise that the decision to use antiplatelet therapy should be individualized based on the degree of vessel stenosis and the presence of additional risk factors or concomitant diseases (e.g., coronary heart disease, cerebrovascular disease, etc.) [68].

According to the Polish Society of Hypertension, as well as recommendations from other international experts, following renal artery catheterization with PTRA, prophylactic doses of low-molecular-weight heparin should be administered for 1–7 days post procedure. Additionally, for the ensuing 3–6 months, the use of acetylsalicylic acid at a dosage of 1 mg/kg body weight per day is recommended [53].

## 9. Conclusions

Renovascular hypertension and mid-aortic syndrome are important causes of arterial hypertension in developmental-age patients. They can occur in the youngest children and also in adolescents. This form of arterial hypertension should be thought of, especially if the patient has severe hypertension, hypertension-mediated organ damage, and abnormalities in ionogram and blood gasometry. The most common cause of RAS in the pediatric population in our part of the world is fibromuscular dysplasia. It is important to remember the possible inflammatory etiology (primarily Takayasu arteritis) and genetic causes (the so-called syndromic forms of RAS and MAS), such as in the course of neurofibromatosis or Williams syndrome. The diagnostic procedure is multistage and includes ultrasound, CT, or MRI scanning, with the final diagnosis made by classical arteriography, which is often also the treatment of choice. Therapeutic management requires an individualized approach and an experienced multi-specialist team.

## Figures and Tables

**Table 1:** Causes of renal artery stenosis in children [1,3,5,13,14,15].

Non-inflammatory causes include: Fibromuscular dysplasia; Mid-aortic Syndrome; Renal artery aneurysm (RAA); Arteriovenous fistula; Segmental Arterial Mediolysis. Inflammatory causes include: Takayasu Arteritis; Kawasaki Disease; Polyarteritis Nodosa; Other systemic vasculitides. Syndromic causes include: Neurofibromatosis Type 1; Tuberous Sclerosis Complex; Williams syndrome; Marfan syndrome; Alagille syndrome; Turner syndrome; Grange syndrome; Congenital rubella; Adenosine deaminase deficiency; Loeys–Dietz syndrome; Myhre syndrome. Localized tissue damage causes include: Trauma; Radiation. Extra-luminal causes include: Compression by tumors such as Wilms’ tumor, Neuroblastoma, and other tumors Compression by diaphragmatic crura; Compression by cysts (autosomal dominant and autosomal recessive polycystic kidney disease); Tuberculosis of renal artery. Intra-luminal narrowing causes include: I. Post-thrombolytic: Catheter-related thromboembolic disease; Umbilical artery catheterization; Hypercoagulable states, e.g., nephrotic syndrome, membranous nephropathy, and antiphospholipid syndrome. II. Other: Idiopathic infantile arterial calcification.Surgical causes include: Transplant renal artery stenosis. Idiopathic

**Table 2:** Genetic causes of RAS and MAS [4,13,20,21,22,23,24,25,26,27,28,29,30,31,32,33,34,35,36].

Clinical Manifestations	Mutation	Syndrome
RAS, MAS, facial abnormalities, growth failure, mental retardation, cardiac defects, hypercalcemia, and aortic stenosis	del(7q11.3)	Williams syndrome
RAS, MAS, intrarenal stenosis, triangular facies, cholestasis, cardiac defects, vertebral abnormalities, embryotoxon posterior, mental retardation, and hypogonadism	JAG1, NOTCH2	Alagille syndrome
RAS, MAS, cafe-au-lait spots, neurofibromas, optic pathway glioma, bilateral high frequency hearing loss, and bone changes	NF1	Neurofibromatosis Type 1
RAS, MAS, skin, bone, renal, cardiac, liver, and ocular lesions, and epilepsy	TSC 1, TSC 2	Tuberous Sclerosis Complex
RAS, MAS, growth failure, primary ovarian failure, and cardiac defects	45XO	Turner syndrome
RAS, skin hyperelasticity, joint laxity, bruising, delayed wound healing, ocular, and cardiovascular defects	COL5A1, COL5A2, TNXB, ADAMTS2, COL3A1, COL1A1, COL1A2, PLOD1, FKBP14 and others	Ehlers–Danlos syndrome
RAS, tall stature, scoliosis, arachnodactyly, enlarged aorta, and dislocation of the lens	FBN1	Marfan syndrome
RAS, stenoses or occlusions of various arteries, bone fragility, brachydactyly, syndactyly, and cardiac defects	YYA1P1	Grange syndrome
RAS, aortic aneurysms,, aneurysms of other vessels, hypertelorism, abnormal uvula, cleft palate, and cardiac defects	TGFBR1, TGFBR2, SMAD2, SMAD3, TGFB2, TGFB3	Loeys–Dietz syndrome
RAS, short stature, facial abnormalities, deafness, mental retardation, muscular hypertrophy, joint limitations, and skeletal abnormalities,	SMAD4	Myhre syndrome

Abbreviations: RAS—Renal artery stenosis, MAS—Mid-aortic syndrome.

**Table 3:** Sensitivity and specificity of various methods of radiological imaging of RAS in children [3,45,46,47].

Specificity	Sensitivity	Radiological Imaging
100%	100%	Renal angiography
83–100%	28–88%	Doppler ultrasound
70–81%	84–88%	Computed tomographic angiography (CTA)
63–100%	63–80%	Magnetic resonance angiography (MRA)

**Table 4:** Normal values of blood flow parameters in the renal arteries according to Polish Ultrasound Society guidelines [47] (* only if Vmax values in abdominal aorta are correct).

Normal Value	Parameter
100 cm/s *	PSV (peak systolic velocity) in artery trunk
0.8–1.0	RAR (renal aortic ratio) in artery trunk
≤70 ms	AT (acceleration time) in intrarenal branches
0.5–0.8	RI (resistance index) in intrarenal branches
0.78–1.33	PI (pulsatility index) in intrarenal branches
>3 m/s^2^	AI (acceleration index) in intrarenal branches

**Table 5:** Pharmacological treatment of RAS and MAS according to the Polish Society of Hypertension 49).

Bilateral Renal Artery Stenosis *	Unilateral Renal Artery Stenosis
Diuretics Dihydropyridine calcium channel blockers Beta-blockers Alpha-blockers Centrally acting imidazoline receptor agonists	Angiotensin-converting enzyme (ACE) inhibitors Angiotensin II receptor blockers (ARB) Dihydropyridine calcium channel blockers Beta-blockers Alpha-blockers Centrally acting imidazoline receptor agonists

* The use of RAAS blockade in cases of bilateral renal artery stenosis or renal artery stenosis in the solitary kidney is absolutely contraindicated.
